# The Novel Role of Platelet-Activating Factor in Protecting Mice against Lipopolysaccharide-Induced Endotoxic Shock

**DOI:** 10.1371/journal.pone.0006503

**Published:** 2009-08-04

**Authors:** Young-Il Jeong, In Duk Jung, Chang-Min Lee, Jeong Hyun Chang, Sung Hak Chun, Kyung Tae Noh, Soo kyung Jeong, Yong Kyoo Shin, Won Suk Lee, Mi Sun Kang, Sang-Yull Lee, Jae-Dong Lee, Yeong-Min Park

**Affiliations:** 1 Department of Microbiology, College of Natural Science, Pusan National University, Geumjeong-Gu, Busan, Korea; 2 Department of Microbiology and Immunology and National Research Laboratory of Dendritic Cell Differentiation & Regulation, Medical Research Institute, College of Medicine, Pusan National University, Seo-gu, Busan, Korea; 3 Department of Pharmacology, College of Medicine, Pusan National University, Seo-gu, Busan, Korea; 4 Department of Clinical Laboratory Science, Daegu Haany University, College of Health & Therapy, Gyeongsangbuk-do, Gyeongsan, Korea; 5 Department of biochemistry, College of Medicine, Pusan National University, Seo-gu, Busan, Korea; 6 Department of Pharmacology, College of Medicine, Chung-ang University, Seoul, Korea; University of Sheffield, United Kingdom

## Abstract

**Background:**

Platelet-activating factor (PAF) has been long believed to be associated with many pathophysiological processes during septic shock. Here we present novel activities for PAF in protecting mice against LPS-mediated endotoxic shock.

**Principal Findings:**

*In vivo* PAF treatment immediately after LPS challenge markedly improved the survival rate against mortality from endotoxic shock. Administration of PAF prominently attenuated LPS-induced organ injury, including profound hypotension, excessive polymorphonuclear neutrophil infiltration, and severe multiple organ failure. In addition, PAF treatment protects against LPS-induced lymphocytes apoptosis. These protective effects of PAF was correlated with significantly decreases in the production of the inflammatory mediators such as TNF-α, IL-1β, IL-12, and IFN-γ, while increasing production of the anti-inflammatory cytokine IL-10 *in vivo* and *in vitro*.

**Conclusions:**

Taken together, these results suggest that PAF may protect mice against endotoxic shock via a complex mechanism involving modulation of inflammatory and anti-inflammatory mediators.

## Introduction

Sepsis is a serious and complex clinical syndrome caused by an overly active host response to infection. sepsis develops in 750,000 people annually, with more than 210,000 cases resulting in death in the United States alone[1]. Under normal conditions, in response to microbial challenge, an immunocompetent host initiates an immediate robust response to constrain and clear the pathogen. However, if the infection is not controlled and spreads beyond the local site, the systemic inflammatory response becomes hyperactive. This pervasive immune response often results in such detrimental complications as multiple organ failure, profound hypotension, and immune paralysis, all of which contribute to the high mortality observed in severe sepsis.

Lipopolysaccharide (LPS) is responsible for initiating this process by inducing the uncontrolled release of proinflammatory mediators from immune cells, particularly monocytes and macrophages. Major proinflammatory cytokines produced during an infection, including tumor necrosis factor (TNF)-α, interleukin (IL)-1β, interferon (IFN-γ), and IL-12, play a prominent roles in defective activation of the host immune response and sepsis-induced tissue injury [2]–[5].

Platelet-activating factor (PAF; 1-O-alkyl-2-(R)-acetyl-sn-glyceryl-3-phosphonocholine) is a potent phospholipid mediator of many leukocyte functions with diverse biological activities [6], [7]. In addition to its role as a physiological mediator, PAF has been associated with the pathology of endotoxic shock. Further, previous studies show that the blood PAF level increased during endotoxemia and that the administration of PAF antagonists in animals protects them from the deleterious effects of endotoxin[8], [9]. For this reason it was postulated that an agent which antagonizes PAF activity may have therapeutic value. However, clinical trials involving administration of PAF receptor (PAF-R) antagonists failed to demonstrate efficacy in diseases such as septic shock, asthma, and pancreatitis [10]. Moreover, experimental studies involving PAF antagonists have produced conflicting results, with some showing improvement in hemodynamic profile and survival, while others not showing any significant differences at all [10]. Interestingly, Walterscheid et al. [11] suggested that PAF plays a critical role in systemic immune suppression induced by the environmental immunotoxin, UV radiation. However, despite extensive investigation, the precise role of PAF among a lot of inflammatory mediators in the development of sepsis still remains largely unknown. In this report, we demonstrate novel pathophylsiological activities of PAF in LPS-induced endotoxemia. PAF provided marked therapeutic activity concomitant with early downmodulation of proinflammatory cytokines and induction of the antiinflammatory cytokine IL-10.

## Results

### PAF protects mice from LPS-induced endotoxemia

**Figure 1:**
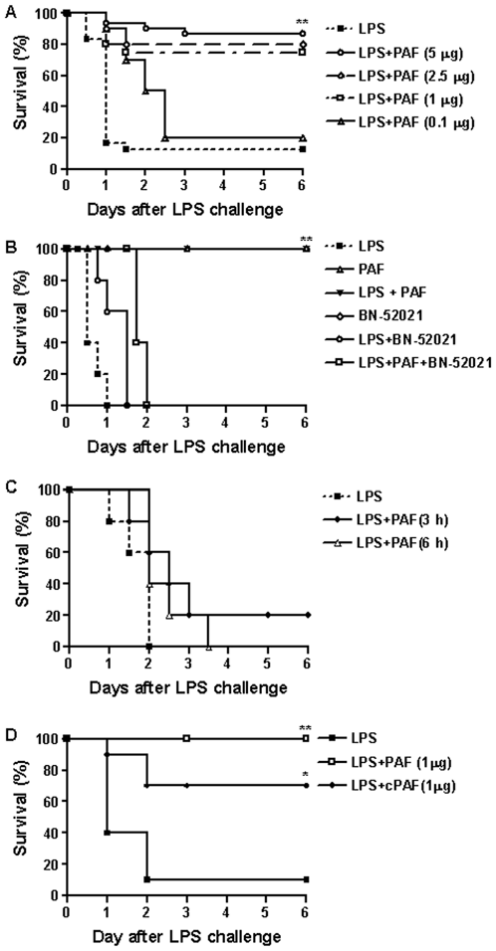
Effect of PAF on the survival rate of LPS-induced endotoxemic mice. (A) Survival of mice i.p. with varying doses of PAF (0.1 to 5 µg/mouse) following LPS challenge (20 mg/kg,) was monitored for 6 d. Shown is one of six experiments with similar results. (B) Treatment with PAF-R antagonist, BN-52021 (80 µg/mouse, i.p), blocked the protective effect of PAF (2.5 µg/mouse) against lethality. (C) Delayed treatment of PAF (5 µg/mouse) had no protective effect against LPS-induced lethality. (D) Effect of cPAF (1 µg/mouse) on survival. N = 8–10 mice per group. *P<0.05; **P<0.001 compared with LPS challenge.

To assess the importance of PAF in protection against endotoxemia, a well-established experimental animal model for endotoxic shock was used. BALB/c mice injected intraperitoneally with a lethal dose LPS (20 mg/kg) nearly died within 36 h of challenge (Figure S1). To determine whether PAF could improve survival in these mice, animals were injected intraperitoneally with vehicle alone or varying doses of PAF immediately after LPS challenge and then monitored for 6 d. PAF significantly improved the mortality of these mice in a concentration-dependent manner starting at the 1 µg dose (Figure 1A).

Next, we examined whether inhibition of PAF-R activation by BN-52021, a PAF-R specific antagonist, could directly affect the protective effects of PAF. In these experiments, animals were treated with vehicle alone, BN-52021, PAF, both compounds immediately after LPS challenge. As controls, mice were also administered with BN-52021 or PAF alone without LPS challenge. Although the mortality of endotoxemic mice that received BN-52021 were slightly delayed compared to those treated with vehicle alone, treatment with the PAF-R antagonist blocked the protective effects of PAF against LPS-induced lethality (Figure 1B).

To evaluate the efficacy of therapeutic treatment (postchallenge) of PAF in preventing of mortality, mice were administered PAF 3 h and 6 h after LPS challenge. A kinetic study revealed that PAF-mediated protection of mice form lethal endotoxemia was substantially reduced when PAF treatment was delayed up to 3 h post-LPS injection (Figure 1C).

Reports have shown that PAF activity is abolished by PAF acetylhydrolase[12]. Carbamyl-PAF (cPAF), an analogue of PAF, is more metabolically stable and capable of maintaining PAF activity for longer durations. Therefore, we assessed the effect of sustained PAF activity on mortality in LPS-induced endotoxemic mice by administering identical doses of cPAF (1 µg/mouse) or PAF (1 µg/mouse) immediately after LPS challenge. There was no difference between the numbers of mice that developed endotoxemia in LPS-induced endotoxemic mice group administrated with PAF and cPAF (0 of 10 vs. 3 of 10, respectively; *p*>0.05). Therefore, cPAF administration was also resistant to LPS-induced lethality (Figure 1D).

### Alteration of LPS-induced inflammatory cytokine production by PAF treatment

**Figure 2:**
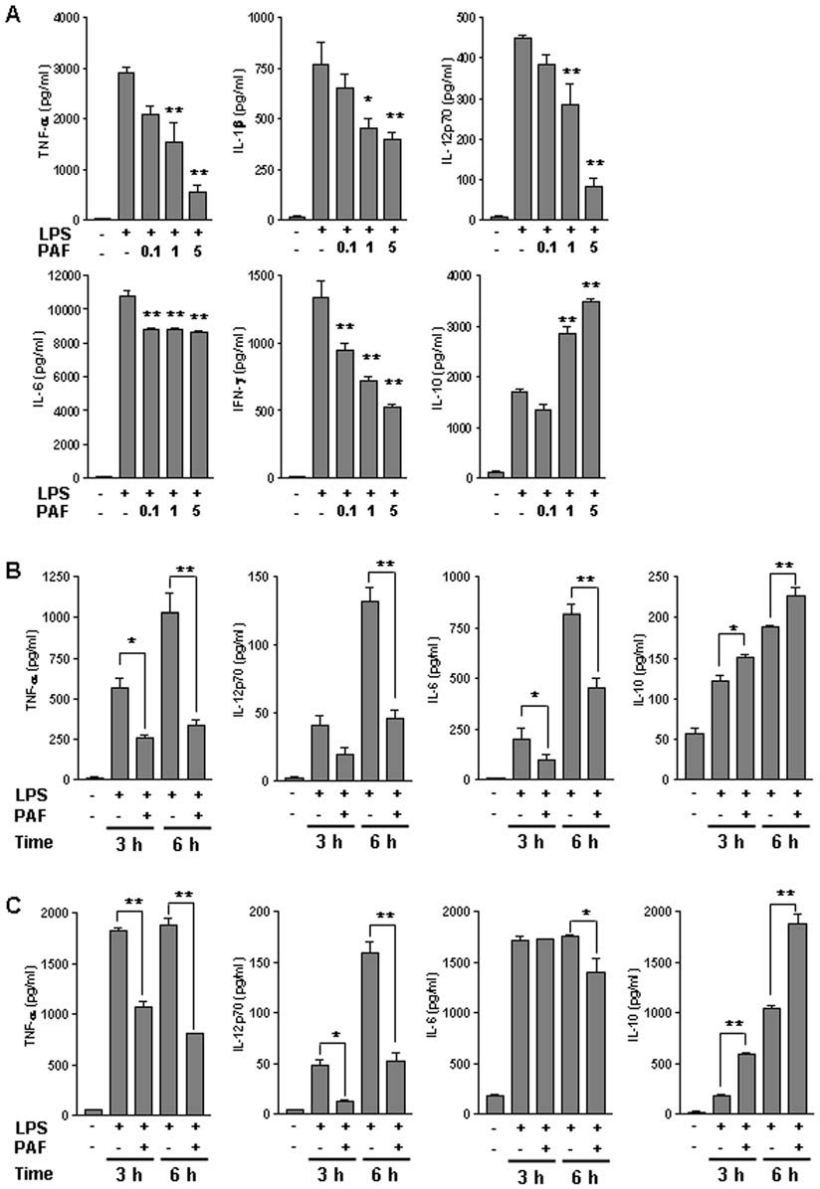
Effects of PAF on LPS-induced inflammatory cytokine production. (A) Mice were injected with vehicle alone (PBS containing 0.25% BSA), or LPS (20 mg/kg, i.p.), or PAF (0.1–5 µg/mouse) immediately following LPS challenge. Blood was collected at 2 h or 6 h (for INF-γ measurement) after injection. PAF treatment altered the serum level of different cytokines in response to LPS in a dose-dependent manner. The mean±SEM values shown represent three independent experiments. *P<0.05; **P<0.001 compared with LPS challenge. (B) Peritoneal macrophages were isolated 3 d following i.p. injection with 2 ml of 4% thioglycollate. Macrophages were then incubated with LPS alone (100 ng/ml) or a combination of LPS and PAF (0.4 µM) for 3 h or 6 h. (C) BMDCs were generated from murine bone marrow cells as described in the Materials and Methods section. BMDCs were stimulated with LPS(100 ng/ml) in the presence of PAF (0.4 µM) for the indicated times. Cytokines concentrations in culture supernatants were measured in triplicate by ELISA. The mean±SEM values shown represent three independent experiments. *P<0.05; **P<0.001.

Endotoxic shock is mediated by an excessive production of proinflammatory cytokines such as TNF-α, IL-1β, IL-6, IL-12 and IFN-γ[13]–[15]. To demonstrate the effect of PAF on LPS-induced inflammatory cytokines production, serum levels of these cytokines following PAF administration in LPS-challenged mice were analyzed. At the 2 h time point, high concentrations of proinflammatory cytokines including IL-12p70, IL-6, IL-1β, and TNF-α were detected in sera. IFN-γ was also significantly increased 6 h after LPS challenge. However, the levels of these cytokines were reduced significantly by PAF treatment in a dose-dependent manner. Interestingly, IL-10, an anti-inflammatory cytokine, was notably elevated by PAF treatment in LPS-challenged mice (Figure 2A)

Macrophages and DCs are a major source of many inflammatory cytokines in immune response. As such, we investigated whether PAF could directly suppress cytokines production in LPS-stimulated macrophages and DCs. PAF treatment of LPS-stimulated peritoneal macrophages remarkable changes in certain cytokine levels. For example, TNF-α, IL-6, and IL-12p70 were decreased, whereas IL-10 was increased substantially compared to that in macrophages stimulated by LPS alone (Figure 2B). Similar to the results obtained from macrophages, TNF-α and IL-12p70 production induced by PAF treatment were decreased, whereas IL-10 was increased significantly compared to that in bone-marrow derived DCs (BMDCs) compared to LPS stimulation alone (Figure 2C). We already identified that PAF-R expression in DCs was induced by LPS or PAF (Figure S2). Unlike macrophages, however, IL-6 was decreased marginally at 6 h (Figure 2C).

### Administration of PAF attenuated LPS-induced organ injury

**Figure 3:**
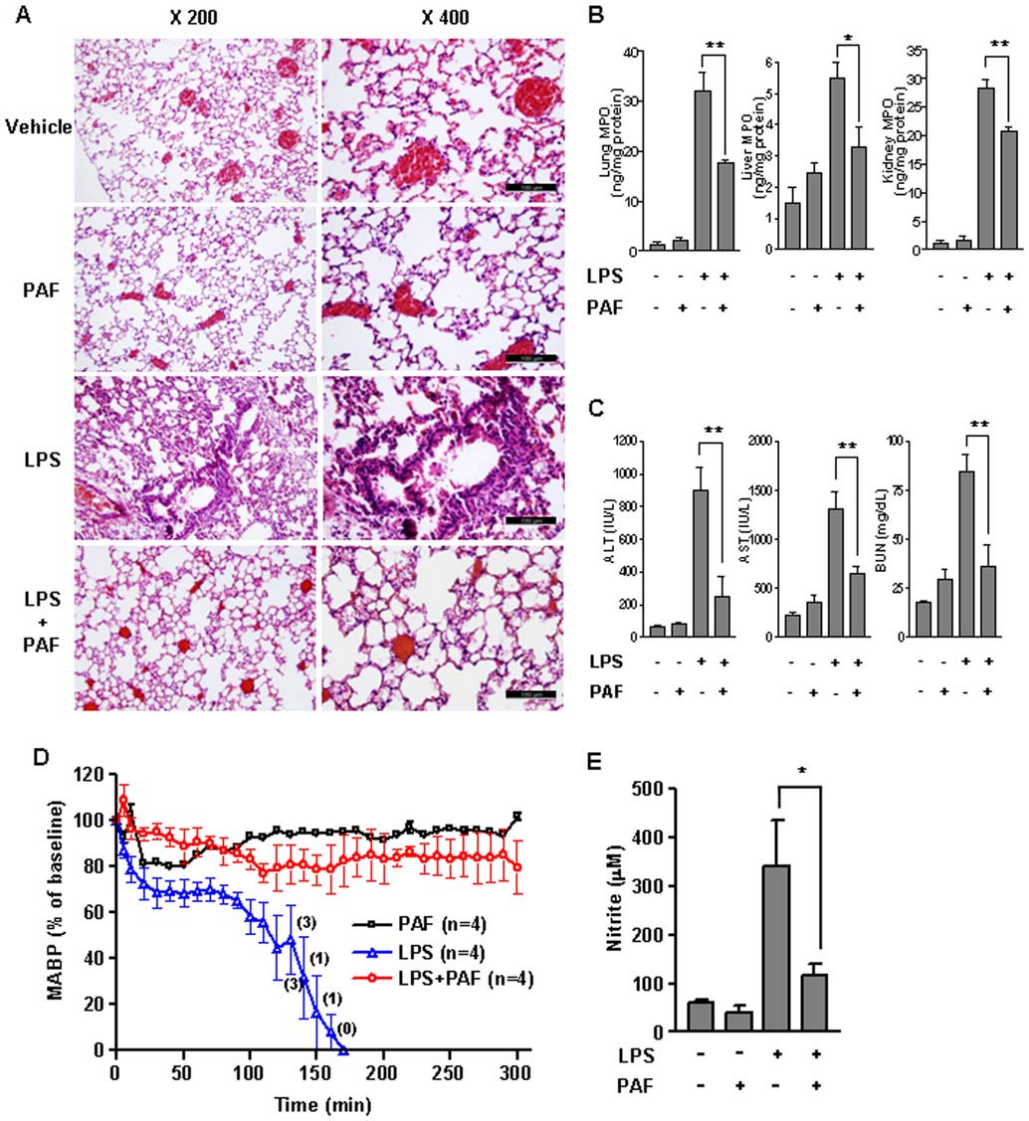
Effect of PAF treatment on organ injury induced by LPS. (A) Lung sections, as well as sera, were obtained from mice 20 h after treatment with vehicle alone, PAF (5 µg/mouse), LPS (10 mg/kg) or LPS plus PAF. Lungs were perfused and fixed with formalin, and sections of lung were stained with hematoxylin and eosin. Shown are representative images of lung sections from each group of mice. (B) The MPO level in the liver, lung, and kidney from each group of mice was assessed 20 h post-injection. (C) The amounts of AST, ALT, and BUN in the sera were measured. The mean±SEM values shown represent three independent experiments. *P<0.05; **P<0.001. (D) Changes in MABP were measured in animals injected with PAF (5 µg/mouse, i.p.), LPS (10 mg/kg, i.p.), or PAF plus LPS. The data points indicate the mean MABP of mice for the 10 min. Results are expressed as a percentage of baseline±SEM. The baseline was set to the mean value over a 10 min period before injection at time 0. The basal MABP of mice were 76.2±5.7 mmHg. Numbers in parentheses are the numbers of animals alive at the indicated times. MABP of dead mice was taken to be zero. (E) PAF inhibits nitric oxide production in LPS-challenged mice. Blood samples were collected 20 h after treatment with vehicle alone, PAF (5 µg/mouse), LPS (10 mg/kg) or LPS plus PAF, and serum nitrite levels were measured by chemiluminescence assay. Data are presented as the mean±SEM of five independent experiments. *P<0.001 compared with LPS-challenged mice group.

Because polymorphonuclear neutrophils (PMNs) infiltration into the major organs such as the lung and liver significantly correlates with the severity of inflammation and is a hallmark of endotoxemia, the effect of PAF treatment on PMN infiltration on these tissues was examined histologically. The lungs of mice injected with PAF alone appeared normal with no observable differences compared to vehicle-treated mice. Mice injected with LPS alone, however, exhibited massive of PMN infiltration into interstitial spaces, marked thickening of the alveolar septa, and pulmonary edema in lung tissue (Figure S3). However, these changes were significantly attenuated in endotoxemic mice treated with PAF (Figure 3A). Similar to the lung, PAF treatment reduced PMN infiltration in the liver (Figure S4). Myeloperoxidase (MPO) is abundant in azurophilic granules of PMNs and its expression level is often used as a measure of PMN recruitment. To quantify the degree of PMN infiltration into lung and liver tissue, MPO activity was assessed. Consistent with the histological changes observed in these tissues, the LPS-mediated increase in MPO level were decreased following PAF treatment, indicating attenuated PMN recruitment (Figure 3B).

Since endotoxemia frequently causes life-threatening inflammatory condition that involves multiple organ injury and dysfunction[16], we examined the effect of PAF administration on LPS-induced organ damages by measuring serum levels ALT and AST, which reveal liver function, and BUN, measurement of renal function. Serum ALT and AST levels in mice injected with both PAF and LPS was lower than those in LPS-challenged mice. In addition, the LPS-induced BUN level was significantly reduced by PAF (Figure 3C). In mice treated with vehicle or PAF alone, liver and renal function tests were substantially unchanged. These results indicated that LPS-initiated organ injury was conspicuously ameliorated by PAF administration.

Hypotension is a clinical characteristic of severe sepsis and plays an important role in the pathophysiology of septic shock and multiorgan failure syndrome. To assess effect of PAF on the regulation of vasculature function during LPS-induced endotoxemia, we measured the mean arterial blood pressure (MABP) in mice with this condition. Although intraperitoneal injection of PAF alone initially demonstrated a potent hypotensive effect, the MABP gradually returned to normal within 50 min post-injection. In contrast to the rapid drop observed during endotoxic shock, the drop in MABP of endotoxemic mice injected with PAF was delayed and sustained at near normal levels for at least 5 h (Figure 3D). Nitric oxide, a major mediator of hypotension, was also analyzed in blood collected 20 h after administration of vehicle alone, PAF, LPS or PAF plus LPS. While serum nitrite levels were elevated with LPS challenge alone, these levels were decreased appreciably when mice were also treated with PAF (Figure 3E). These results strongly indicate that PAF treatment attenuates LPS-induced organ injury.

### PAF protects against lymphocytes apoptosis

**Figure 4:**
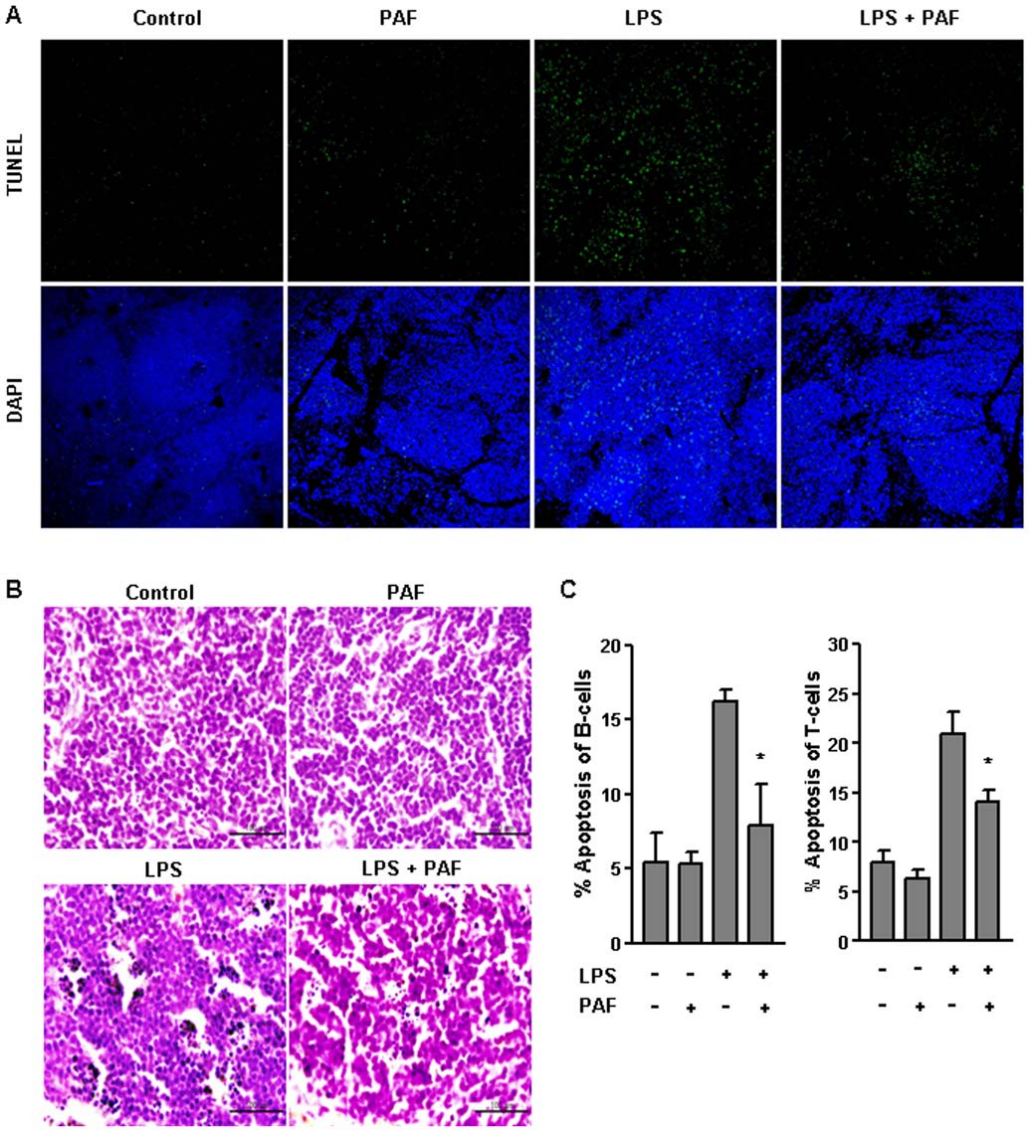
Exogenous PAF prevents lymphocyte apoptosis in LPS-induced endoxemia. BALB/c mice (7 wk old, n = 3) were injected with either vehicle alone, PAF (5 µg/mouse, i.p.), LPS (10 mg/kg, i.p.), or both and sacrificed after 20 h. (A) Apoptotic cells fluorescently labeled using the TUNEL assay. Fixed paraffin-embedded spleens were labeled using the TUNEL assay to detect apoptotic cells and then examined by laser scanning confocal microscopy at magnifications of×100. (B) Hematoxylin and eosin staining, which illustrated the morphological changes (compacted and fragmented nuclei) in cells undergoing apoptosis, revealed splenocytes from endotoxic mice underwent extensive apoptosis while spleen from endotoxic mice treated with PAF did not. (C) Flow cytometric analysis of apoptosis. Splenocytes from each group of mice were examined for apoptosis by flow cytometry using Annexin V staining. T and B cells were distinguished by staining with PE-conjugated anti-CD3 and anti-CD19 Abs, respectively. LPS-induced endotoxemia increased apoptosis in both cell types, while PAF protected them against endotoxemia-induced cell death. The mean±SEM values shown represent three or four independent experiments. *P<0.05 compared with LPS-challenged mice group.

Recent several studies have demonstrated that lymphocyte apoptosis may be detrimental during sepsis due to the depletion of lymphocytes that essential for defense against invading microorganisms [5], [17], [18]. We examined that the effect of PAF on lymphocytes apoptosis induced by endotoxemia. LPS challenged mice showed a dramatic increase in TUNEL positive cells that was significantly reduced with PAF treatment (Figure 4A). The protective effect of PAF on endotoxin-induced lymphocyte apoptosis was also confirmed by hematoxylin and eosin staining, which illustrated the morphological changes in cells undergoing apoptosis in LPS challenged mice. Consistent with previous reports[18], apoptotic cells in the spleen of LPS-challenged mice possessed small and compact nuclei (pyknosis) with multiple nuclear fragments (apoptotic bodies). However, cells from PAF administered-mice had less nuclear contraction and fragmentation (Figure 4B). Flow cytometry with Annexin V staining also demonstrated that a lethal dose of LPS caused a marked increase in T and B cells apoptosis. However, consistent with the findings obtained from the TUNEL and hematoxylin and eosin staining, PAF administration prevented apoptosis in these cells (Figure 4C). Finally, no apparent apoptosis was observed in the spleen of mice administered with PAF alone.

## Discussion

This study demonstrated the protective effect of exogenous PAF administration against LPS-induced endotoxemia and identified the molecular mechanisms involved in this biological process. Contrary to previous pharmacologic reports concerning the role of PAF in inflammation, our results demonstrate that mice treated with PAF acquired resistance to LPS-induced endotoxic shock, and that this effect can be blocked by the PAF-R antagonist BN-52021 (Figure 1A, B). Although no therapeutic activity was observed until PAF treatment was delayed to 6 h after LPS challenge, treatment with PAF before or immediately after a lethal LPS dose protected mice against endotoxic death (Figure 1C). These results challenge the current paradigm of PAF as an important mediator of sepsis, which is based on the concept that septic shock results from an uncontrolled inflammatory response. For many years, studies on the biological effects of PAF as a potent inflammatory mediator were mainly been focused on the activation of cells involved in inflammation. Thus, many clinical trials for severe sepsis attempted to inhibit the action of PAF with a variety of PAF-R antagonists. Although septic animal models exhibit beneficial effects as a result of PAF antagonist treatment, clinical studies on patients with sepsis do not display similar outcome. Because the dose of PAF-R antagonists which inhibit endotoxin-induced sepsis are typically more than 10-fold higher than those for PAF released during sepsis, it is suggests that protective effect of PAF antagonist may be related in non-specific inhibition [19]. Study using PAF-R deficient mice further verified these points. Ishii S *et al* [19] observed no significant differences in lethality and production of inflammatory cytokines during endotoxic shock between wild-type and PAF-R-deficient mice, implying that PAF is not essential for endotoxic shock development. Recently, Walterscheid *et al* [11] provided evidence for a novel immunoreglatory role for PAF, which, in addition to being a sensor for cellular damage, can activate immune suppressive mechanisms. Our present findings further support this hypothesis, suggesting that the beneficial effect of exogenous PAF occurred primarily by interference with the cascade of events ultimately leading to the onset of severe endotoxin shock.

Sepsis is just one example of a pathologic condition associated with a cytokine storm, the excessive and sustained production of numerous cytokines by immune cells. Much evidence derived from studies in animal and in human systems show that highly elevated levels of proinflammatory cytokines contribute to high mortality by septic shock [2], [4]. Our results demonstrate that, in addition to protecting against endotoxin-mediated high mortality, PAF induces remarkable changes in the production level of cytokines in response to LPS. In particular, two distinct patterns were observed. First, in LPS-induced endotoxemic mice, PAF administration resulted in prominent decrease in the production of circulating proinflammatory cytokines, including TNF-α, IL-1β, IL-12p70, and IFN-γ (Figure 2). Second, PAF administration significantly increased production of the compensatory anti-inflammatory cytokine IL-10 (Figure 2). Because anti-inflammatory cytokines are released as a regulatory mechanism in septic shock and several studies using animal models of sepsis have demonstrated that recombinant IL-10 has a protective effect against mortality and proinflammatory cytokine production [20]–[23], it is possible that augmented IL-10 production by PAF may contribute to a compensatory response during endotoxin shock. And also, we observed that PAF-mediated protection of mice from lethal endotoxemia could be blocked by prior administration of neutralizing anti-IL-10 antibodies, but not by an isotype control antibody (J.Y.I., unpublished observation). These results implicate that IL-10, as one of mechanisms involved in the capacity of PAF to protect mice from LPS-induced toxic shock, confers partially on systemic immune suppression.

Exaggerated proinflammatory cytokines activity can result in symptoms of septic shock. Specially, TNF-α and IL-1β contributed to the increase in the number of infiltrating neutrophils which play a critical role in bacterial clearance[24]. Our data demonstrate that after LPS challenge, massive PMN infiltration in the lung and liver was promoted (Figure 3A). In addition, the level of lung, liver, kidney MPO was increased (Figure 3B). Interestingly, histological examination of liver and lung sections showed that PMN accumulation in PAF-administrated mice were significantly lower than those in LPS-challenged mice. Correspondingly, there was a trend toward a decrease in MPO levels in the lung, liver, kidney of PAF-treated endotoxemic mice. By analyzing serum biochemical parameters that assess liver damage (AST/ALT) and renal function (BUN), we found that PAF treatment significantly reduced levels of tissue damage following LPS administration (Figure 3C). Severe hypotension is an important hallmark of endotoxic shock and has been linked to iNOS expression and excessive NO production. Coincident with alleviation of LPS-induced hypotension, PAF treatment markedly reduced NO production (Figure 3D, E). These findings indicate that the protective effect of PAF against LPS lethality results from a marked decrease in all characteristic of severe tissue injury in LPS-induced endotoxemia. Extensive lymphocyte apoptosis is critical pathogenic event in sepsis [5], [17], [18]. As such, it was noteworthy to investigate whether exogenous PAF treatment in endotoxemic mice exerts an inhibitory effect on apoptosis of immune effector cells. Our *in vivo* studies demonstrate that PAF inhibits T and B lymphocyte apoptosis in LPS-induced endotoxemic mice (Figure 4), indicating that survival in endotoxin mice may be improved by PAF treatment.

Collectively, our findings demonstrate the immunosuppressive effects of exogenous PAF in containing the host immune response to bacterial products. Present findings of unexpected pathophysiological PAF activities in the LPS-mediated endotoxic shock suggest that the role of PAF in regulating the immune response may be more complex beyond its established role as a pro-inflammatory mediator. We speculate that PAF may be a significant pharmacological target for treatment of patients with endotoxic shock.

## Materials and Methods

### Animals

The animal protocol used in this study has been reviewed by the Pusan National University–Institutional Animal Care and Use Committee (PNU-IACUC) on their ethical procedures and scientific care, and it has been approved (Approval Number PNU-2008-0001).BALB/c mice at 7∼8 wk of age were purchased from the Korean Institute of Chemistry Technology (Daejeon, Korea) and were used. The animals were housed in a specific pathogen-free environment within our animal facility and used in accordance with the institutional guidelines for animal care.

### Reagents

PAF, carbamyl-PAF (cPAF), and BN-52021 were purchased from Sigma-Aldrich.

### LPS-induced endotoxemia model

For LPS-mediated endotoxemia models, mice were injected i.p. with the designated dose of LPS (*E.coli* O127:B8, Sigma-Aldrich) dissolved in PBS containing 0.25% bovine serum albumin (BSA). The general conditions and mortality were recorded for up to 6 d after injection to ensure that no additional late deaths occurred. The designated doses of PAF were administered i.p. immediately after LPS (20 mg/kg), unless otherwise stated. PBS containing 0.25% BSA was used as a control treatment.

### Isolation of peritoneal macrophages and dendritic cells

To isolate elicited peritoneal macrophages, mice were injected i.p. (2 ml/mouse) with 4% Brewer thioglycollate medium (Sigma-Aldrich) and peritoneal exudate cells were harvested 3 d later. DCs were generated from murine BM cells as described by previously [25] with some modifications. In brief, BM was flushed from the tibiae and femurs of Balb/c and depleted of red cells with ammonium chloride. The cells were plated in six-well culture plates (10^6^ cells/ml; 3 ml/well) in RPMI-1640 medium (Hyclone) plus 10% heat-inactivated FBS (Hyclone) and 20 ng/ml recombinant murine granulocyte-macrophage colony- stimulating factor (rm GM-CSF) at 37°C, 5% CO_2_. On day 6, 80% or more of the nonadherent cells expressed CD11c.

### Measurement of Cytokines

Serum and culture supernatants of peritoneal macrophages or BMDCs were collected and assayed for various cytokine levels using enzyme-linked immunosorbent assay (ELISA). ELISA kits were purchased from Assay Designs and R&D Systems.

### Clinical Chemistry

Serum levels of aspartate transaminase (AST), alanine transaminase (ALT), and blood urea nitrogen (BUN) activity were measured by Laboratory Medicine, Clinical Pathology at Pusan National University Hospital using Automatic Hematology Analyzer (Hitachi modular system, Hitachi Ltd., Tokyo, Japan).

### Measurement of PMN infiltration

Lungs were first perfused with 10% formalin at constant distending pressure of 25 cm H_2_O for 10 min. Liver and lung were excised from the animals, and then placed in 10% formalin overnight at 4°C. And then, these were tissues embedded in paraffin and 4-µM sections were prepared and stained with hematoxylin and eosin.

### Myeloperoxidase (MPO) estimation

Neutrophil sequestration in lung, liver, and kidney was quantified by measuring tissue MPO activity. Tissue Samples for MPO analysis were frozen in liquid nitrogen immediately after removal from the animal and were thawed, homogenized in following lysis buffer: 200 mM NaCl, 5 mM EDTA, 10 mM Tris, 10% glycerol, 1 mM PMSF, 1 mg/ml leupeptide and 28 mg/ml aprotinine (pH 7.4). Centrifuge samples twice (6,000 g at 4°C for 15 min) to avoid contamination of cell debri and the cleared supernatant was used for the MPO assay. MPO activities were measured using a mouse MPO ELISA kit (Hycult biotechnology b.v., Uden, The Netherlands), according to the manufacturer's instructions. Protein concentration in the supernatants was measured by protein dye binding assay (protein assay; Bio-Rad Laboratories). The levels of MPO in organ extracts were expressed as nanogram per milligram of protein.

### Measurement of mean arterial blood pressure (MABP)

Under urethane anesthesia, we performed cannulation of the carotid artery, and then, MABP of mice were monitored by laser-Doppler flowmetry (FLO-N1, Omegawave, Tokyo, Japan).

### Flow cytometry

Spleens were gently glass-ground to dissociate the cells. Cells were then washed twice in PBS. Residual red blood cells were lysed by hypotonic lysis in ice-cold ammonium chloride. The cells were resuspended in PBS and the desired fluorescent indicators added. The degree of apoptotic cell death was quantified using a commercially available annexin V-FITC detection apoptosis kit I (BD PharMingen, San Diego, CA, USA). Mouse T and B cells were identified by fluorescently labeled mouse monoclonal antibodies specific for the desired cell subtype (BD PharMingen). Identification of apoptotic cells by annexin V staining and cell phenotyping were done simultaneously using flow cytometry on a FACS-Calibur (Becton Dickinson). Cell debris was electronically gated out, based on forward light scatter.

### TUNEL Assay

To detect apoptotic cells, fixed paraffin-embedded spleens were labeled by the terminal deoxynucleotidyl transferase dUTP nick-end labelling TUNEL assay using a commercially-available kit from Upstate (Lake Placid, NY, USA) and were then examined by laser scanning confocal microscopy.

### Statistics

Experiments were repeated at least three times with consistent results. Prism 4.0 (GraphPad Software) was used for tests. A two-tailed, unpaired Student's t-test with 95% confidence bounds was used for statistical analysis unless otherwise indicated.

## Supporting Information

Figure S1 The survival rate of LPS-induced endotoxemic mice.
(0.01 MB PDF)

Figure S2 PAF-R expression is substantially induced by stimulating with PAF or LPS
(0.06 MB PDF)

Figure S3 LPS-induced neutrophils infiltration into lung
(0.06 MB PDF)

Figure S4 Administration of PAF attenuated LPS-induced neutrophils infiltration into liver
(0.15 MB PDF)
