# Supplementary material for: The Novel Role of Platelet-Activating Factor in Protecting Mice against Lipopolysaccharide-Induced Endotoxic Shock
Source: PLoS One. 2009 Aug 4;4(8):e6503. doi: 10.1371/journal.pone.0006503 (PMC2714981; doi:10.1371/journal.pone.0006503)
Supplement: Figure S1 — The survival rate of LPS-induced endotoxemic mice. (0.01 MB PDF) [file pone.0006503.s001.pdf]

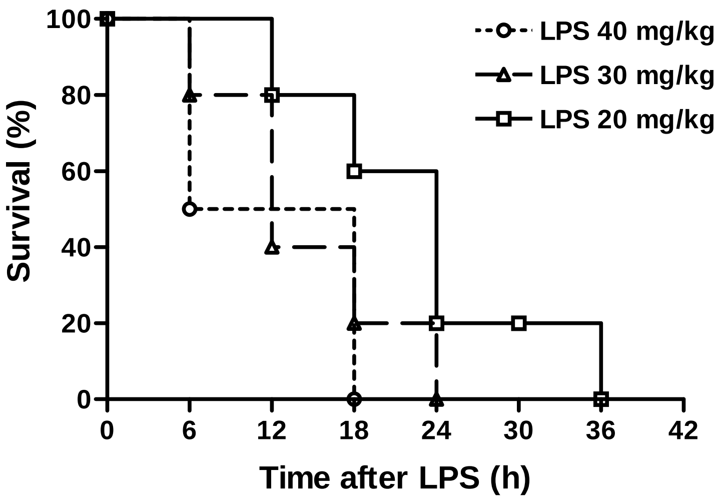

Supplementary Figure 1 The survival rate of LPS-induced endotoxemic mice. Survival of mice i.p. with varying doses of following LPS challenge (40 to 20 mg/kg,) was monitored for 42 h.
