# Supplementary material for: The Novel Role of Platelet-Activating Factor in Protecting Mice against Lipopolysaccharide-Induced Endotoxic Shock
Source: PLoS One. 2009 Aug 4;4(8):e6503. doi: 10.1371/journal.pone.0006503 (PMC2714981; doi:10.1371/journal.pone.0006503)
Supplement: Figure S2 — PAF-R expression is substantially induced by stimulating with PAF or LPS (0.06 MB PDF) [file pone.0006503.s002.pdf]

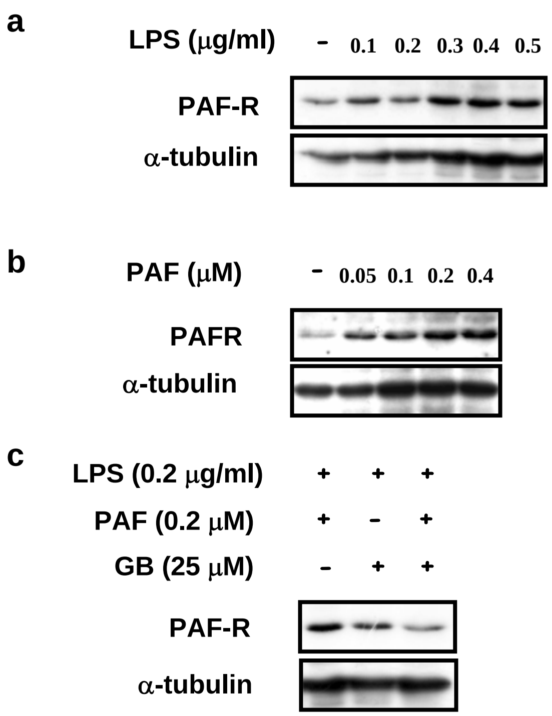

Supplementary Figure 2 PAF-R expression is substantially induced by stimulating with PAF or LPS. **(a, b)** BMDCs were incubated with varying doses of LPS (100 to 500 ng/ml) or PAF (0.05 μM to 0.4 μM) for 18 h. **(c)** BMDCs were treated in combination with PAF, LPS, or BN 52021(BN) at indicated dose for 18 h. Lysates were prepared and analyzed by Western blot with Abs specific to PAF-R. This data is representative of three independent experiments.
