# Supplementary material for: The Novel Role of Platelet-Activating Factor in Protecting Mice against Lipopolysaccharide-Induced Endotoxic Shock
Source: PLoS One. 2009 Aug 4;4(8):e6503. doi: 10.1371/journal.pone.0006503 (PMC2714981; doi:10.1371/journal.pone.0006503)
Supplement: Figure S3 — LPS-induced neutrophils infiltration into lung (0.06 MB PDF) [file pone.0006503.s003.pdf]

X 1000

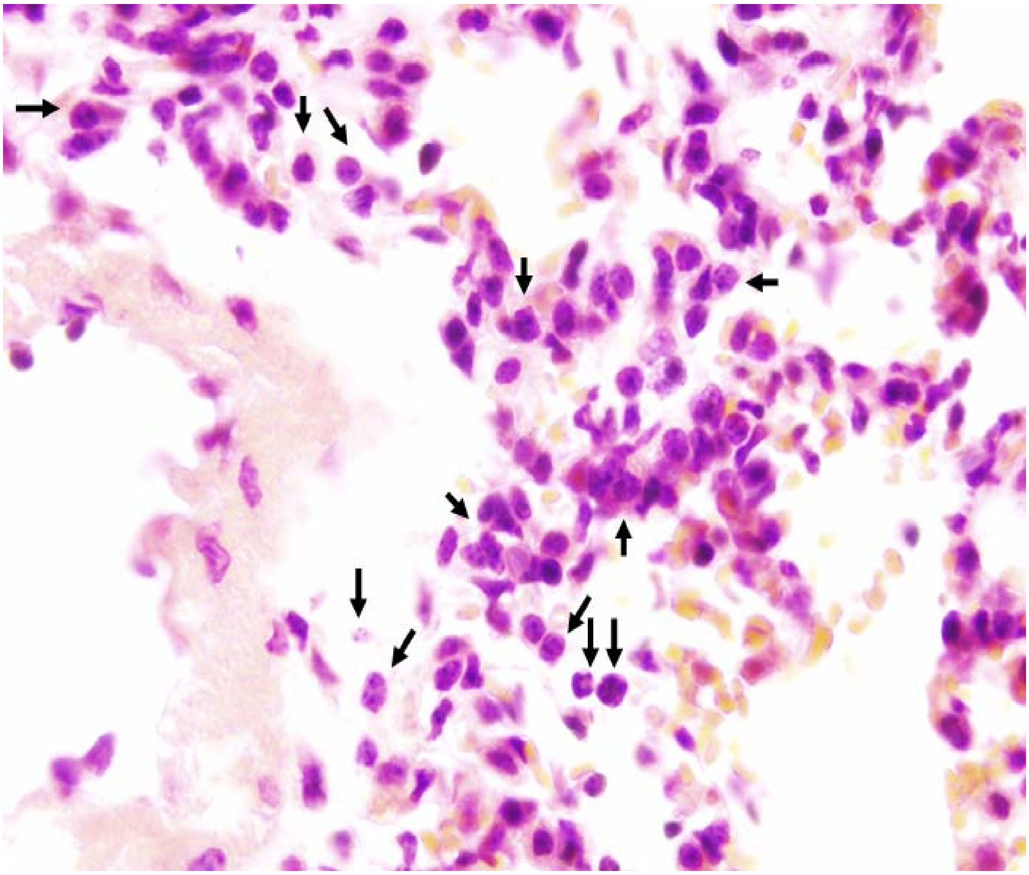

Supplementary Figure 3 LPS-induced neutrophils infiltration into lung. lung sections were obtained from mice 20 h after treatment with LPS (10 mg/kg). Sections of lung were stained with hematoxylin and eosin. In LPS-challenged mice, marked accumulation of neutrophils was observed. Arrows indicate the infiltrating neutrophils.
