# Supplementary material for: The Novel Role of Platelet-Activating Factor in Protecting Mice against Lipopolysaccharide-Induced Endotoxic Shock
Source: PLoS One. 2009 Aug 4;4(8):e6503. doi: 10.1371/journal.pone.0006503 (PMC2714981; doi:10.1371/journal.pone.0006503)
Supplement: Figure S4 — Administration of PAF attenuated LPS-induced neutrophils infiltration into liver (0.15 MB PDF) [file pone.0006503.s004.pdf]

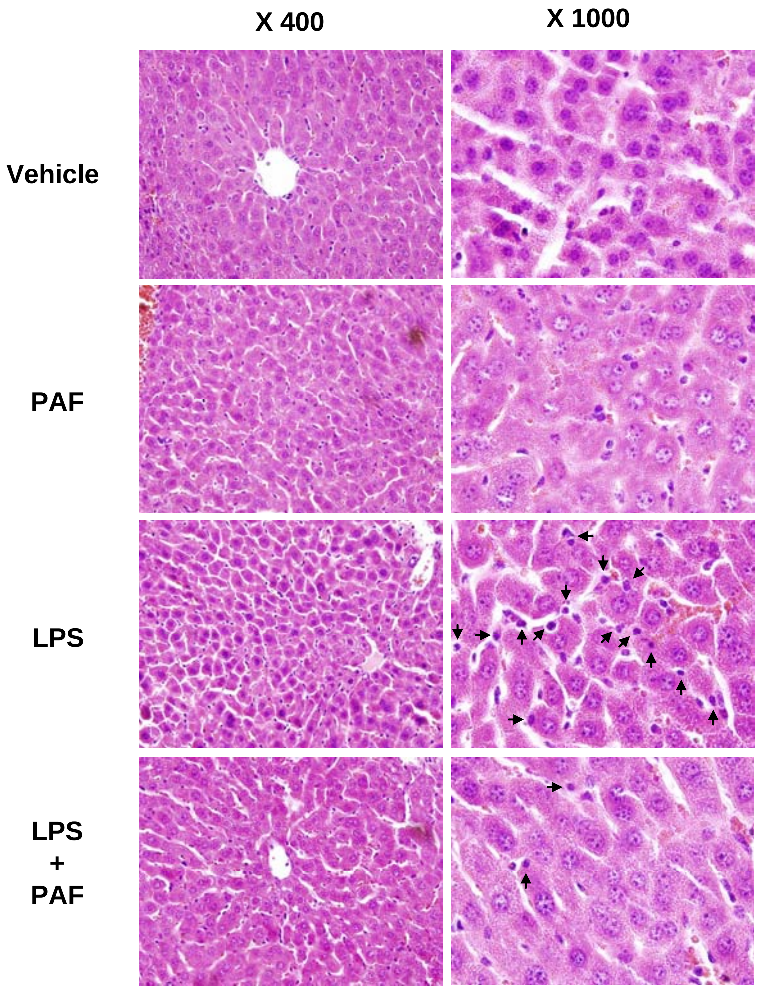


**Supplementary Figure 4** Administration of PAF attenuated LPS-induced neutrophils infiltration into liver. liver sections were obtained from mice 20 h after treatment with vehicle alone, PAF (5 μg/mouse), LPS (10 mg/kg) or LPS plus PAF. Sections of liver were stained with hematoxylin and eosin. Shown are representative images of  liver sections from each group of mice. In LPS-challenged mice, marked accumulation of neutrophils was observed. In contrast, few neutrophils in the lung and liver of LPS-induced endotoxemic mice administered with PAF can be seen. Arrows indicate the infiltrating neutrophils.
